# Supplementary material for: Survival analysis of under-five mortality and associated risk factors using survival analysis approaches
Source: PLOS Glob Public Health. 2025 Sep 12;5(9):e0005179. doi: 10.1371/journal.pgph.0005179 (PMC12431492; doi:10.1371/journal.pgph.0005179)
Supplement: Attachment — Submitted filename: survival analysis review.docx [file pgph.0005179.s001.docx]

1. **Abstract:** Needs some clarity in background
2. **Introduction:** Does not give any background regarding the under 5 mortality and its associated factors, Needs uniform citation style
3. **Sampling method:** better if explained using a flow chart
4. **Predictors:** variable names need to be proper instead of mentioning it as it is in E-xcell sheet
5. **Results:** Under Kaplan-Meier curves and log-rank test there is statement mentioned as outlined in Section 2.2.2 – this need clarity
6. **Figure 1:** Spell check to be done on legends
7. Use similar abbreviations everywhere
8. Results from the semi-parametric Cox-PH model: what is section 3.1
9. Checking violation the Cox-PH model assumption: maternal wealth is not mentioned in adjusted cox-ph, what is section 2.3.1.2
10. **Table 2,3:** variables need to be properly named
11. **Table 4:** explanation is not correct
12. **Discussion:** Repetition from introduction and methodology noted. Authors insightful thinking and reasoning is not reflected
13. Conclusion does not answer the objective
