# Supplementary material for: Survival analysis of under-five mortality and associated risk factors using survival analysis approaches
Source: PLOS Glob Public Health. 2025 Sep 12;5(9):e0005179. doi: 10.1371/journal.pgph.0005179 (PMC12431492; doi:10.1371/journal.pgph.0005179)
Supplement: Attachment — Submitted filename: Response to Reviewers.docx [file pgph.0005179.s003.docx]

Dear Editor,

We would like to thank the reviewers for the many useful and insightful comments. We have now

revised the manuscript substantially. We trust that the revised manuscript now meets your required

standards for publication. Please find below our responses to the comments. All changes made due to the reviewers’ comments are highlighted in red text with track changes inside the revised manuscript.

**Edito**r:

***Comment 1.1****: Please note that PLOS Global Public Health has specific guidelines on code sharing for submissions in which author-generated code underpins the findings in the manuscript. In these cases, all author-generated code must be made available without restrictions upon publication of the work. Please review our guidelines at https://journals.plos.org/globalpublichealth/s/materials-and-software-sharing#loc-sharing-code and ensure that your code is shared in a way that follows best practice and facilitates reproducibility and reuse.*

**Response 1.1:** Thank you for these comments. In response, data analyses were carried out using STATA and R softwares and codes are available upon request from the corresponding author.

***Comment 1.2:*** *We have noticed that you have cited Table 5 in the manuscript file but there are no corresponding tables in the manuscript. Please amend your manuscript to include this table, noting that tables should not be uploaded as individual files.*

**Response 1.2:** We thank the Editor this important observation. The text “Tables 4 and 5” has been revised to “Table 4” (**Page 15, line 21**)

***Comment 1.3:*** *Please note that your Data Availability Statement is currently missing [he repository name and the DOI/accession number of each dataset. If your manuscript is accepted for publication, you will be asked to provide these details on a very short timeline. We therefore suggest that you provide this information now, though we will not hold up the peer review process if you are unable.*

**Response 1.3:** We thank the Editor for this useful suggestion. In response, we have included the repository name and the DOI/accession number of each dataset. The original dataset ([Working-data.dta](https://zenodo.org/api/records/16538777/draft/files/Working-data.dta/content)) for creating the survival outcome, survival time, predictors, fitting the univariable and multivariable semi-parametric models and checking Cox-PH assumptions can be found at: <https://doi.org/10.5281/zenodo.16538777>. The dataset ([Working-data2.csv](https://zenodo.org/api/records/16538777/draft/files/Working-data2.csv/content)) for fitting the extended Cox-PH model can be found at: <https://doi.org/10.5281/zenodo.16538777>. The repository name is Datasets for Semi-Parameteric and Extended Cox-Proportional Hazard Models (Page 24, lines 20-26).

**Reviewer #1:**

***Comment 1.1:*** *Whether the predictors considered for the model were selected on basis of previous literature or statistics?*

**Response 1.1:** We thank the reviewer for this question. In response, predictors were selected on the basis of previous literature (**Page 6, lines 5-6**).

***Comment 1.2:*** *reduction in mortality risk with receipt of 2–7 tetanus vaccinations is an interesting finding but looks underexplored and unsupported. It could be because of poor health access to mothers who received only one dose of or high-risk pregnancies. Also is it pertaining to death during neonatal period or during infancy?*

**Response 1.2:** We thank the reviewer for this valuable observation regarding the finding on the reduction in under-five mortality risk associated with receipt of 2–7 tetanus vaccinations. In response, this pertains to death until 59 months. The observed association between receipt of multiple tetanus toxoid doses and reduced mortality warrants cautious interpretation. Biologically, repeated doses sustain maternal antibodies that protect neonates via passive immunity during the vulnerable neonatal period, consistent with WHO recommendations for up to five lifetime doses. However, this finding may also reflect differences in health service access, as mothers receiving multiple doses likely have better antenatal care and lower overall risk, while those with only one dose may be more underserved. The effect is most likely driven by protection against neonatal deaths from tetanus, though indirect benefits may extend into infancy. Further analysis using cause-specific mortality data is needed to clarify timing and potential confounding factors (**Discussion**, **Page 21, lines 27-30 and page 22, lines 1-5**).

***Comment 1.3:*** *Caesarean sections were linked to a 63% increase in under-five mortality. Whether it is with respect to emergency Caesarean sections or elective Caesarean sections. Such findings are likely to be influenced by confounding factors which should be considered in analysis and require discussion.*

**Response 1.3:** We thank the reviewer these important comments. In response, we agree with the reviewer that “*Such findings are likely to be influenced by confounding factors which should be considered in analysis and require discussion.*“ To address potential confounding, we adjusted for key maternal and child factors available in the dataset, including maternal age, parity, birthweight (proxied by size at birth), and antenatal care attendance. However, we acknowledge that residual confounding is possible due to unmeasured factors such as obstetric complications, comorbidities, or quality of perinatal care. The observed association may reflect underlying risk profiles leading to emergency caesarean section rather than an adverse effect of caesarean delivery (**Page 22, lines 14-21**).

In the DHS dataset, cesarean delivery is recorded by asking whether birth as delivered by cesarean section, with a simple Yes/No response coded as binary, and no additional information on the indication, timing, or whether the procedure was elective or emergency; thus, differentiating elective from emergency cesarean sections is not possible using standard GDHS datasets unless linked to additional clinical or facility-level data. This a key limitation of the DHS dataset (**Limitation**, **Page 24, lines 1-11**).

***Comment 1.4:*** *It is stated that average and large-sized infants showing a substantially increased risk of mortality. This is in contrast to previous research and needs discussion whether it was associated with other complications in neonatal period.*

**Response 1.4:** We appreciate the reviewer’s insightful observation. We agree that our finding of higher mortality risk among average- and large-sized infants contrasts with much of the existing literature, where small size at birth is typically associated with increased mortality risk. One possible explanation is that large or average-sized infants in our dataset may include those affected by complications such as birth asphyxia, shoulder dystocia, or maternal conditions (e.g., gestational diabetes or obstructed labor), which are known risk factors for neonatal morbidity and mortality. Additionally, our size-at-birth variable is based on maternal recall rather than measured birthweight, which may introduce misclassification bias. We have added these points with supporting references to the discussion section, highlighting the need for further research using clinically measured birthweight and information on perinatal complications to clarify this unexpected association (**Page 22, lines 25-30 and page 23, lines 1-3**).

***Comment 1.5****: Some specific key findings for recommendations could be summarised in discussion.*

**Response 1.5:** We thank the reviewer for this important recommendation. In response, we have summarized specific key findings for recommendations in the discussion section as “There is the need to strengthen antenatal and neonatal care to reduce preterm births and manage high-risk pregnancies, expanding maternal immunization programs, and improving postnatal follow-up for caesarean deliveries. Investments in family planning and reproductive health services are essential to reduce unwanted pregnancies, while strategies to improve maternal education and alleviate socioeconomic disparities can further enhance child survival outcomes. These targeted interventions are critical to sustaining progress toward reducing child mortality and achieving the Sustainable Development Goals.” (**Recommendation, Page 23, lines 22-28**)

**Reviewer #2:**

***Comment 1.1:*** *Abstract: Needs some clarity in background*

**Response 1.1:** We thank the reviewer for these comments. In response, we have revised the abstract’s background as “The risk of under-five mortality serves as a critical indicator of the performance of a country’s healthcare system and is directly aligned with the Sustainable Development Goals (SDG 3.2), which aim to end preventable deaths of newborns and children under five, reducing the rate to at least 25 deaths per 1,000 live births by 2030. Although Ghana has made significant progress in reducing child mortality over recent decades, current levels remain above this global target. Understanding the predictors of under-five mortality risk is therefore essential for developing evidence-based health policies and targeted interventions to accelerate progress toward SDG 3 and improve overall child health outcomes. To identify these predictors, we employed survival modeling techniques. The commonly used Cox-proportional hazards (Cox-PH) model assumes constant covariate effects over time; however, violation of this proportional hazard assumption can result in biased statistical inferences. To address this, we applied the extended Cox-PH model, which accounts for time-varying effects. Data were derived from the 2022 Ghana Demographic and Health Survey (GDHS), collected using a stratified two-stage cluster sampling design. Parameter estimation and analyses were conducted using R version 4.4.3 and STATA/SE 18.0 (StataCorp, TX, USA).“

**Comment 1.2:** *Introduction: Does not give any background regarding the under 5 mortality and its associated factors, Needs uniform citation style.*

**Response 1.2:** We thank the reviewer for this useful observation. In response, we have included background regarding under-five mortality and associated factors in the introduction (**Page 2, lines 19-35 and page 3, lines 1-17**)

***Comment 1.3:*** *Sampling method: better if explained using a flow chart.*

**Response 1.3:** Thank you for this useful suggestion. In response, we have explained the sampling methods using flow-chart in **Fig 1.**

***Comment 1.4:*** *Predictors: variable names need to be proper instead of mentioning it as it is in E-xcell sheet.*

**Response 1.4:** Thank you for this important suggestion. We revised variables names accordingly (**Page 6, lines 1-30 and Page 7, lines 1-5**)

***Comment 1.5:*** *Results: Under Kaplan-Meier curves and log-rank test there is statement mentioned as outlined in Section 2.2.2 – this need clarity.*

**Response 1.5:** We thank the reviewer for this important observation. In response, we have deleted “as outlined in Section 2.2.2” and the text now reads “Similarly, we applied these methods to examine the survival probability of under-five mortality across other potential predictors” (**Page 9, lines 14-16**).

***Comment 1.6****: Figure 1: Spell check to be done on legends.*

**Response 1.6:** Thank you for this important observation. In response, we have revised the Figure 1 legends. Figure 1 is now Figure 2 since the sampling method’s flow-chart is labeled Figure 1.

***Comment 1.7****: Use similar abbreviations everywhere.*

**Response 1.7**: Thank you for this observation. We have revised the entire manuscript to ensure that similar abbreviations are used.

***Comment 1.8:*** *Results from the semi-parametric Cox-PH model: what is section 3.1*

**Response 1.8**: We thank the reviewer for this important observation. In response, we have revised the text to read “The unaHR results aligned with the results from the Kaplan-Meier and log-rank methods” (**Page 10, lines 12-13**).

***Comment 1.8:*** *Checking violation the Cox-PH model assumption: maternal wealth is not mentioned in adjusted cox-ph, what is section 2.3.1.2.*

**Response 1.8**: Thank you for these important observations. In response, we have revised the text to now read “In the multivariable Cox-PH model, the variables sex, twins, gestational period, maternal education, and maternal wealth exhibited violations, as their respective p-values were below 0.05.” (**Page 13, lines 12-14**). We have deleted all the sections citations throughout the manuscript.

***Comment 1.9:*** *Table 2,3: variables need to be properly named.*

**Response 1.9**: Thank you, this suggestion. We have renamed the variables in Tables 2 and 3 for easy readability.

***Comment 2.1:*** *Table 4: explanation is not correct*

**Response 2.1:** We have revised the text to reflect the definition of Table 4.

**Comment 2.2:** Discussion: Repetition from introduction and methodology noted. Authors insightful thinking and reasoning is not reflected.

**Response 2.2:** We thank the reviewer for these comments. In response, we have updated the discussion section to provide deeper insights into the findings, incorporating both policy recommendations and study limitations (**Page 21, lines 27-30 and page 22, lines 1-30 and page 23, lines 1-3**).

***Comment 2.3:*** *Conclusion does not answer the objective.*

**Response 2.3:** We thank the reviewer for this important observation. In response, we have revised the conclusion section to reflect the objective of the study (**Page 23, lines 15-20**)
