# Supplementary material for: Survival analysis of under-five mortality and associated risk factors using survival analysis approaches
Source: PLOS Glob Public Health. 2025 Sep 12;5(9):e0005179. doi: 10.1371/journal.pgph.0005179 (PMC12431492; doi:10.1371/journal.pgph.0005179)
Supplement: Attachment — Submitted filename: Response_to_Reviewers_auresp_2.docx [file pgph.0005179.s004.docx]

Dear Editor,

We would like to thank the reviewers for the many useful and insightful comments. We have now

revised the manuscript substantially. We trust that the revised manuscript now meets your required

standards for publication. Please find below our responses to the comments. All changes made due to the reviewers’ comments are highlighted in red text with track changes inside the revised manuscript.

***Comment 1.1:*** *Inconsistency in Interpretation of Male Sex Risk; Issue: The abstract states that males have a 4% increased risk (aHR = 1.04), but Table 4 shows aHR = 1.04 with 95% CI (1.10, 1.30)—which contradicts; Suggestion: Correct either the abstract or results section. Likely aHR = 1.20 or 1.28 is correct, not 1.04.*

**Response 1.1**: We thank the reviewer for this important observation. In response, the correct estimates should be aHR=1.04 with 95%CI as 1.01, 1.30). We have revised Table 4 **(Page 17-18)** and the results interpretation **(Page 22, lines 1-19).**

***Comment 1.2: E****xaggerated Unadjusted Hazard Ratios; Issue: Many unadjusted HRs (e.g., tetanus vaccination = 40.28) appear biologically implausible. This may stem from overfitting or collinearity; Suggestion: Clarify modeling steps that reduce these inflated values. Include a note on possible bias in unadjusted estimates.*

**Response 1.2:** We appreciate the reviewer’s observation regarding the unusually large unadjusted hazard ratios (e.g., tetanus vaccination = 40.28). In response, we have fitted Bayesian survival and multilevel survival models to address this issue **(Tables 5 and 6)**

***Comments 1.3:*** *Terminology Clarity for Lay Readers; Issue: Some readers may not understand statistical terms like Schoenfeld residuals, time-varying effects, or interaction terms; Suggestion: Include a plain-language box or footnote on these concepts, or refer to online appendices.*

**Response 1.3:** We thank the reviewer for highlighting the need to make statistical terminology understandable to a wider readership. We agree that terms such as Schoenfeld residuals, time-varying effects, and interaction terms may be unfamiliar to non-technical readers. To address this, we have added short clarifying footnotes in the main text at the first mention of these terms in **Page 8.**

***Comment 1.4:*** *Birth Size Findings Require Caution; Issue: The finding that average and large infants have higher mortality risk contradicts most global evidence; Suggestion: Discuss possible recall bias (since birth size is self-reported), misclassification, or non-linear effects. Recommend modelling continuous weight (if available).*

**Response 1.4:** We thank the reviewer for this important observation. We agree that the finding that infants reported as “average” or “large” at birth were associated with higher mortality risk than “small” infants contrasts with much of the existing literature, which generally shows increased risk among smaller newborns. In response, we have included a statement regarding this finding in the discussion section **(Page 26, lines 7-12).**

***Comment 1.5:*** *Missing Data Analysis Not Reported. Nowhere in the manuscript is there mention of missingness pattern, missing data proportion, or imputation strategy. DHS datasets often contain missing values due to non-response or skip patterns, especially for maternal recall variables (e.g., size at birth, pregnancy intention, antenatal visits). Cox models (semi-parametric or extended) assume complete case analysis by default. If missing data are not MCAR (Missing Completely at Random), listwise deletion can introduce bias and reduce statistical power.* ***Suggestions for Revision****: Perform and report a missing data exploration: Proportion and pattern of missingness. Missingness by outcome status (survival/death).* ***Consider using****: Multiple imputation (MI) or inverse probability weighting. Sensitivity analysis to assess robustness under different missingness assumptions. Example language to add: “We conducted Little's MCAR test and found that the data were not MCAR (p < 0.01). Therefore, multiple imputation using chained equations was applied for variables with >5% missingness.”*

**Response 1.5:** We thank the reviewer for highlighting this important point regarding missing data. We agree that, in general, missing data should be carefully addressed using appropriate methods such as multiple imputation under the Missing at Random (MAR) assumption, and sensitivity analyses under Not Missing at Random (NMAR) assumptions (e.g., pattern-mixture, selection, or shared-parameter models) are recommended as best practice. In our study, however, none of the variables from the Ghana Demographic and Health Survey (GDHS) dataset used in the analysis contained missing values (see GDHS dataset at <https://dhsprogram.com/methodology/survey/survey-display-598.cfm>, or available at [Zenodo DOI:10.5281/zenodo.16538777](https://doi.org/10.5281/zenodo.16538777)). Both the outcome (child survival status) and all covariates were complete for all observations. We have now clarified this in the analysis/results section **(Page 10, lines 2-12)**.

***Comment 1.6:*** *No Data Balancing or Class Imbalance Handling. No discussion of the imbalance between the number of deaths and survivals (a common issue in child mortality analysis). Under-five death is typically rare in DHS samples (<10%), which can lead to biased model estimates or unstable hazard ratios. Imbalanced outcomes can: Inflates Type I errors. Make HR estimates unstable or sensitive to outliers. Mislead significance when using standard tests. Report the event rate (death % of total).* ***Consider applying:*** *Case-control reweighting or oversampling methods (e.g., SMOTE for time-to-event outcomes). Penalized Cox models (e.g., Firth’s correction) if data sparsity is evident. Alternatively, use Bayesian survival models for rare-event stabilization.*

**Response 1.6:** We thank the reviewer for these insightful comments regarding the potential impact of outcome imbalance on model estimates. In response, in our dataset, under-five deaths account for less than 10% of the total sample, consistent with the typical rarity of such events in DHS surveys. To address this, we have now conducted Bayesian survival analysis specifically to handle these rare-event outcomes. Hence, we have revised the abstract **(Page 1, lines 27-28)**, introduction **(Page 4, line 28)**, methods **(Page 7, lines 13-16, Page 9, lines 13-21)**, and (**Pages 18-19, Table 5**).

***Comment 1.7:*** *No Strategy to Handle Population Heterogeneity. No attempt to account for unobserved heterogeneity across regions or households (despite DHS being cluster-sampled). There is no use of frailty models, stratified models, or random effects, though such approaches are common in DHS survival analysis. IIgnoring heterogeneity can: Violate independence assumptions. Underestimate standard errors (inflated Type I errors). Miss important subgroup effects.*
**Response 1.7:** We thank the reviewer for this important observation regarding population heterogeneity. Indeed, ignoring unobserved heterogeneity in survival models, especially when using DHS dataset, can bias inference and increase the risk of Type I errors. In response to the reviewer’s comment, we have now extended our analysis to explicitly account for population heterogeneity inherent in the DHS data. Specifically, we fitted a **multilevel survival model** that includes **random effects (frailty terms) at both the cluster and regional levels**. This approach captures unobserved heterogeneity across households and regions, accommodates the hierarchical sampling design of the DHS, and relaxes the independence assumption among individuals within the same cluster. We have accordingly revised the abstract **(Page 1, lines 28-29)**, introduction **(Page 4, lines 28-30)**, method section **(Page 9, lines 23-28)**, and results **(Page 19-20)**
